# Assessing the Influence of Thermocycling on Compressive Strength, Flexural Strength, and Microhardness in Green-Mediated Nanocomposite-Enhanced Glass Ionomer Cement Compared to Traditional Glass Ionomer Cement

**DOI:** 10.7759/cureus.56078

**Published:** 2024-03-13

**Authors:** Bharath Ravi, Jessy Paulraj, Subhabrata Maiti, Rajeshkumar Shanmugam

**Affiliations:** 1 Department of Pedodontics and Preventive Dentistry, Saveetha Dental College and Hospitals, Saveetha Institute of Medical and Technical Sciences, Saveetha University, Chennai, IND; 2 Department of Prosthodontics, Saveetha Dental College and Hospital, Saveetha Institute of Medical and Technical Sciences, Saveetha University, Chennai, IND; 3 Nanobiomedicine Lab, Centre for Global Health Research, Saveetha Medical College and Hospital, Saveetha Institute of Medical and Technical Sciences, Chennai, IND

**Keywords:** green-mediated nanoparticles, microhardness, strength, thermocycling, nanocomposite-modified gic

## Abstract

Background and objective

Glass ionomer cement (GIC), also known as polyalkenoate cement, has been extensively used in dentistry for both luting and restorative purposes. Despite being the first choice for aesthetic restorations due to their chemical bonding ability to teeth, GICs have faced challenges such as low mechanical properties, abrasion resistance, and sensitivity to moisture, leading to the search for improved materials.

This study aims to assess the effects of thermocycling on the compressive, flexural strength, and microhardness of green-mediated nanocomposite-modified GIC in comparison to traditional GIC.

Methodology

Green-mediated nanoparticles, consisting of chitosan, titanium, zirconia, and hydroxyapatite (Ch-Ti-Zr-HA), were synthesized using a one-pot synthesis technique to form nanocomposites. These nanocomposites were then incorporated into GIC specimens in varying concentrations (3%, 5%, and 10%), denoted as Group I, Group II, and Group III, respectively. Group IV served as the control, consisting of conventional GIC. To assess the performance of the novel restorative materials over an extended period, compressive strength, flexural strength, and microhardness were measured before and after thermocycling using a universal material testing machine. Furthermore, scanning electron microscopy (SEM) analysis was carried out following the thermocycling process. The collected data were subjected to statistical analysis through one-way analysis of variance (ANOVA) and paired t-tests.

Results

The findings demonstrated that, in comparison to the control group, both the mean compressive strength and flexural strength, as well as hardness, were notably higher for the 10% and 5% nanocomposite-modified GIC specimens before and after thermocycling (*P *< 0.05). Notably, there was no notable difference observed between the 5% and 10% concentrations (*P* > 0.05). These results suggest that incorporating green-mediated nanocomposites (Ch-Ti-Zr-HA) modified GIC at either 5% or 10% concentration levels leads to improved mechanical properties, indicating their potential as promising alternatives in dental restorative materials.

Conclusions

Based on our findings, it can be inferred that the 10% and 5% concentrations of green-mediated (Ch-Ti-Zr-HA) modified GIC exhibit superior compressive and flexural strength compared to conventional GIC. Additionally, analysis of the scanning electron microscope (SEM) morphology revealed that green-mediated GIC displays smoother surface characteristics in contrast to conventional GIC. These results underscore the potential advantages of utilizing green-mediated nanocomposite-modified GIC in dental applications, suggesting enhanced mechanical properties and surface quality over conventional.

## Introduction

Glass ionomer cement (GIC) exhibits distinctive features such as anti-cariogenic properties and chemical adhesion to tooth structure. However, it also exhibits limitations in clinical use due to limited wear resistance, fracture toughness, and vulnerability to early moisture sensitivity [1,2]. These drawbacks have constrained their application in stress-bearing areas, necessitating further advancement of GIC. Attempts have been undertaken to enhance the strength of GIC by incorporating various filler materials [3-5]. However, concerns regarding the chemical composition and potential toxicity of these additives remain a topic of debate, hindering their market acceptance. Recent research has shown that the inclusion of nanoparticles like hydroxyapatite (HA), produced through soft chemistry processes to create nanoscale particles, has the potential to enhance GIC properties [6]. Additionally, a study by Ibrahim et al. demonstrated that GIC modified with chitosan and TiO_2_ nanopowder exhibits superior mechanical properties [7]. Therefore, this study focuses on synthesizing nanocomposite chitosan, titanium, zirconia, and hydroxyapatite (Ch-Ti-Zr-HA) using green synthesis methods, thereby modifying it with GIC to assess its mechanical properties.

The clinical effectiveness of a dental material is determined by its capacity to withstand the stresses and strains encountered during mastication and routine oral functions. Therefore, strength is of paramount importance when selecting a restorative material, as higher strength is better equipped to withstand deformation and fractures, thus minimizing the risk of failure. Hence, compressive strength and flexural strength are commonly assessed mechanical properties in GIC. Additionally, scanning electron microscopy (SEM) serves as an effective technique for examining the surface characteristics, filler content, size, and interface of restorations. SEM is particularly useful for identifying the types of failures experienced by restorations, as well as surface modifications and wear [8].

When a restorative material is exposed to the oral environment over a prolonged period, various changes can occur in its properties. Additionally, thermal stresses encountered during normal oral functions can disrupt the structure of restorative materials and potentially impact their mechanical properties. Numerous studies have investigated the effects of thermal stresses on restorative materials [9,10]. Therefore, characterizing dental restorative materials using thermocycling provides a better simulation of their clinical service life and enhances understanding of their behavior in such conditions. Accordingly, the objective of this study was to evaluate the impact of green-mediated nanocomposite (Ch-Ti-Zr-HA) modified GIC compared to conventional GIC on mechanical strength (compressive and flexural strength) and microhardness before and after thermocycling. The null hypotheses were that green-mediated nanocomposite-modified GIC would not affect mechanical strength and microhardness compared to conventional GIC before and after thermocycling.

## Materials and methods

Estimation of sample size and study design

Ethical approval was granted, and the study was registered with the research center under the code SRB/SDC/UG-2086/23/PEDO/139. The sample size calculation was conducted using the GPower sample power calculator. It indicated that for a sample power of 0.95 (95% confidence interval) and an effect size of 0.6, each group would need to include 48 samples.

Preparation of green-mediated Ch-Ti-Zr-HA nanoparticles 

Chitosan nanoparticles were prepared by stirring a mixture of 50 mL eucalyptus extract and 50 mL chitosan solution, which consisted of 0.5 g chitosan powder mixed with 0.5 g glacial acetic acid and 49 mL distilled water, using a magnetic stirrer. Next, titanium oxide nanoparticles were synthesized by combining 50 mL neem extract with 50 mL of 50 mM TiO_2_ solution and stirring the mixture with a rotating magnet. Then, zirconium oxide nanoparticles were prepared by adding 50 mL aloe vera extract to 50 mL of 20 nM zirconium oxide solution, continuously stirring the mixture at 340-350 °C with a magnetic stirrer, and allowing it to stand overnight. Lastly, HA nanoparticles were obtained by mixing 50 mL Moringa oleifera extract (1 g) with 50 mL of 0.1 g HA synthesized from eggshell. Orthophosphoric acid, with a molar ratio of 1.67 Ca/P, was added dropwise, stirred, and left to stand overnight.

Preparation of green-mediated Ch-Ti-Zr-HA nanocomposites

The nanocomposites were fabricated using a one-pot synthesis method [11]. The resulting solutions were vigorously stirred at 80 °C for 30 minutes. Subsequently, 1.08 mL of ethanol was introduced into the mixture, followed by vigorous stirring at 80 °C under reflux conditions for 90 minutes. Following the removal of ethanol at 80 °C for 30 minutes, the solution was subjected to lyophilization in a freeze dryer for 48 hours at -92 °C to yield a fine powder. This gentle freeze-drying process aimed to improve the long-term stability of the nanoparticles while retaining their biochemical properties.

Preparation of green-mediated nanocomposite (Ch-Ti-Zr-HA) modified GIC specimen

Green-mediated nanocomposites consisting of Ch-Ti-Zr-HA were integrated into GIC at concentrations of 3% (Group I), 5% (Group II), and 10% (Group III). Additionally, a control group, Group IV, was included, which utilized conventional GIC without any modifications. The powder components of the nanocomposites and the conventional GIC were combined with the polyacrylic acid-based liquid to produce the restorative cement.

Micromorphology analysis

SEM analysis of GIC specimens was modified with green-mediated nanocomposites. Initially, each specimen underwent rinsing with sterile x1 phosphate-buffered saline (PBS). The specimens were soaked in glutaraldehyde (2.5%) in PBS for four hours at 48 °C, followed by 10-minute rinses with x1 PBS. Next, the specimens underwent treatment with osmium tetroxide (1%) for an hour, followed by 10-minute rinses in x1 PBS and a final rinse with distilled water. Dehydration was accomplished through a graded ethanol process at room temperature. The dehydrated specimens were then dried, mounted onto SEM aluminum stubs, and coated with gold-palladium for 100 seconds under vacuum conditions. SEM was utilized for examination, operating at an accelerating voltage of 10 KV and capturing representative areas at 33,000 magnifications. Additionally, one sample from each group underwent a similar process, including dehydration and gold sputtering, and was examined at x1,000 magnification using SEM for more detailed analysis. This comprehensive protocol ensured a thorough evaluation of the structural characteristics of the specimens.

Thermocycling 

**Figure 1:**
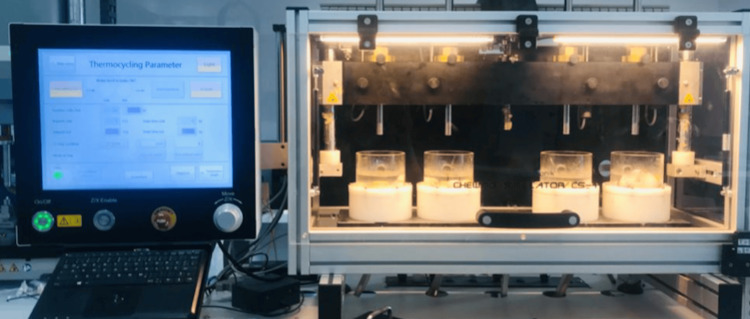
Samples undergoing immersion within a thermocycler.

During the research, samples were subjected to thermocycling using a Lab Thermostatic Bath within a thermocycler. The water baths were consistently set at 55°C throughout the thermocycling process, which consisted of 30,000 cycles at this temperature, each lasting 15 seconds. Continuous monitoring was carried out to ensure the stability of both temperature and dwell time. After the thermocycling process was completed, the specimens were examined for any visible changes, and the collected data, which included assessments of structural integrity, surface morphology, and material properties were analyzed (Figure 1).

Assessment of compressive strength

**Figure 2:**
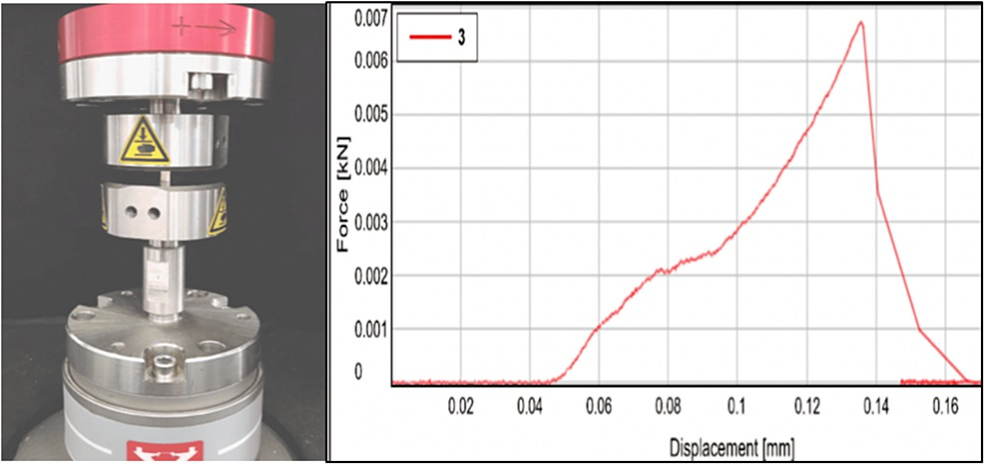
Compressive strength analysis performed with UTM. UTM, universal testing machine

The assessment of compressive strength for the specimens was conducted at a standard temperature of 23 ± 1 °C following the guidelines outlined in ISO 9917-1:2007. Cylindrical molds (4.0 mm x 6.0 mm ) were prepared, yielding approximately 12 specimens for each group. After introducing the materials into the molds and ensuring a level surface, the specimens were left in the molds for an hour. Subsequently, they were removed and immersed in deionized water for 24 hours to assess compressive strength. Any specimens displaying deformation or voids were excluded. The specimens were vertically aligned in the universal testing machine (UTM-Instron, E3000). Compressive strength was assessed by loading the specimens until fracture at a crosshead speed of 1 mm/minute and fracture load was calculated (Figure 2).

Assessment of flexural strength

**Figure 3:**
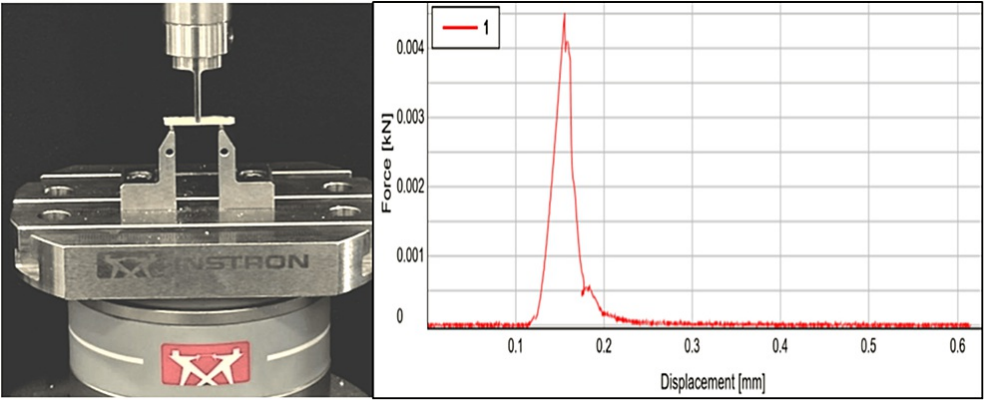
Flexural strength analysis performed with UTM. UTM, universal testing machine

Flexural strength was evaluated as per ISO 9917-2 guidelines where rectangular specimens (25 × 2 × 2 mm³) were prepared by mixing the materials as per the manufacturer's instructions. These specimens were then placed in the molds and gently compressed using a mylar strip. After 10 minutes of setting, the specimens were removed from the molds and placed in a chamber with elevated humidity at 37 °C for 24 hours. Defective or voided specimens were discarded. The dimensions of the specimens were measured using a digital micrometer with a precision of 0.001 mm. Next, the specimens were subjected to a three-point bending test using a universal testing machine (UTM-Instron, E3000) to assess their flexural strength. The flexural strength of each specimen was then calculated (Figure 3).

Assessment of microhardness 

**Figure 4:**
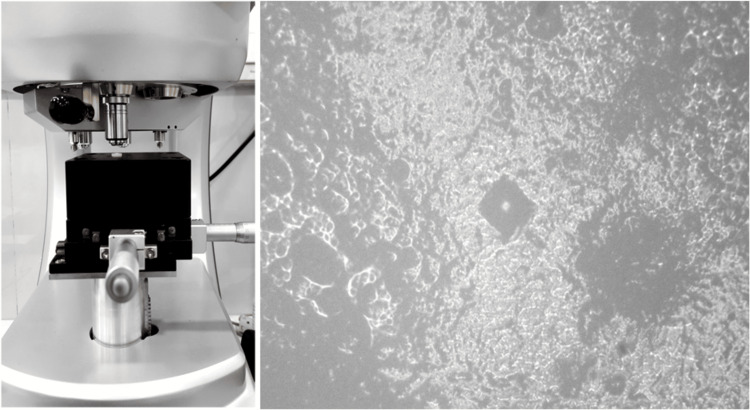
Microhardness analysis performed using a Vickers Hardness Tester.

In Vickers microhardness testing, a pyramidal diamond with a 136° facing angle is pressed onto a surface under a specified load for a set duration. The Shimadzu HMV-G31DT Micro Vickers Hardness Tester was used in this study. The applied force for the microhardness test was HV0.3 (2.942 N), and the indenter was held in place for 20 seconds. The Vickers microhardness number is calculated by measuring the width of the imprint left on the surface after the diamond has been removed (Figure 4).

Statistical test 

The data collection process was initiated with the utilization of Google Sheets, followed by the importation of data into IBM SPSS Statistics for Windows, Version 26.0 (IBM Corp., Armonk, NY) for statistical analysis. The primary objective was to assess statistical significance at a predetermined level of α = 0.05. To analyze all parameters before and after thermocycling, a one-way ANOVA was conducted. Subsequently, Tukey's post hoc analysis was utilized to detect any notable discrepancies among the different groups. For a more detailed examination of the contrast between pre- and post-simulation values, a paired t-test was performed.

## Results

Micromorphological characteristics 

**Figure 5:**
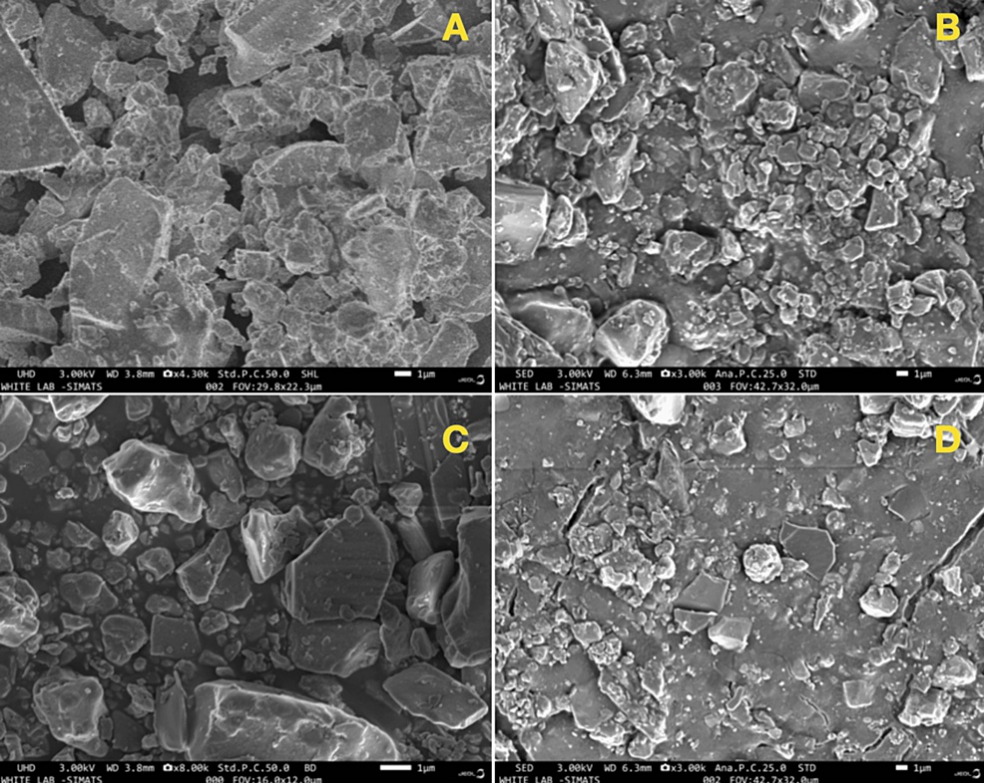
Representative scanning electron microscopy (SEM) images of the glass ionomer cement (GIC) surface after thermocycling. (A) Group I (3%); (B) Group II (5%); (C) Group III (10%); (D) Group IV (control).

Analysis of GIC using SEM after nanomodification at different concentrations reveals noticeable changes in surface morphology. Gradual increments in nanomodifier concentration show a clear improvement in microstructure. SEM images at lower concentrations display a granular surface with scattered nanosized particles, suggesting subtle modifications to the GIC matrix. Conversely, higher concentrations of nanomodifiers induce a more pronounced effect, resulting in a denser and uniformly distributed arrangement of nanoparticles. This translates into a smoother and more compact microstructure, as evident in SEM images. The heightened surface morphology with escalating concentrations implies a direct relationship between nanomodifier concentration and structural modifications within GIC. Post-thermocycling images reveal more cracks in the control group, whereas the modified groups exhibit a denser morphology. These SEM-based observations highlight the tangible impact of varying nanomaterial concentrations on GIC surface characteristics. This insight underscores the potential improvement in mechanical properties and overall performance, positioning SEM as an essential tool for comprehending the microscale effects of nanomodification on the material (Figure 5).

Compressive strength, flexural strength, and microhardness

A significant difference was observed in compressive strength, flexural strength, and microhardness between pre- and post-thermocycling conditions (*P *= 0.001). In the pre-thermocycling phase, the group with a 10% concentration exhibited the highest compressive strength (196.45 ± 2.83) and flexural strength (32.67 ± 4.76), followed by the 5%, 3%, and control groups, respectively. Following thermocycling, there was a consistent reduction in compressive strength and flexural strength across all groups, maintaining the same order, with the 10% group exhibiting the highest compressive strength (196.41 ± 0.83) and flexural strength (31.88 ± 5.5), followed by the 5%, 3%, and control groups. Increased concentrations of nanocomposites demonstrated markedly improved microhardness, with the highest observed at 10% (before 50.29 ± 1.35; after 52.06 ± 4.22), followed by 5%, 3%, and the control group (Table 1).

**Table 1:** Comparison of strength and microhardness of green-mediated nanocomposite (Ch-Ti-Zr-HA) modified GIC and conventional GIC before and after thermocycling. ^*^Statistically significant value of *P* <0.05. The *P*-value was derived from a paired *t*-test GIC, glass ionomer cement; SD, standard deviation; Ch-Ti-Zr-HA, chitosan, titanium, zirconia, and hydroxyapatite

Parameter	Groups	Before thermocycling (Mean ± SD)	After thermocycling (Mean ± SD)	Mean difference	*t*-value	*P*-value
Compressive strength	3%	178.57 ± 0.10	174.84 ± 6.94	3.73 ± 6.95	8.15	0.090
	5%	188.91 ± 2.90	190.10 ± 3.11	1.18 ± 3.91	1.30	0.317
	10%	196.45 ± 2.83	196.41 ± 0.83	0.041 ± 2.99	1.96	0.962
	Control	167.85 ± 1.02	156.89 ± 14.33	10.96 ± 14.08	19.91	0.021^*^
Flexural strength	3%	28.10 ± 2.32	26.42 ± 1.31	1.67 ± 2.89	2.00	0.70
	5%	30.96 ± 1.65	29.79 ± 1.89	1.16 ± 3.02	1.33	0.208
	10%	32.67 ± 4.76	31.88 ± 5.55	0.79 ± 7.60	0.361	0.725
	Control	16.86 ± 0.19	10.87 ± 0.92	5.98 ± 0.92	22.45	0.001^*^
Microhardness	3%	45.54 ± 1.84	46.75 ± 3.74	1.20 ± 3.22	1.29	0.220
	5%	48.54 ± 0.86	49.84 ± 3.54	1.30 ± 4.09	1.10	0.295
	10%	50.29 ± 1.35	52.06 ± 4.22	1.76 ± 4.55	1.34	0.206
	Control	41.61 ± 0.94	42.32 ± 2.36	0.70 ± 2.48	0.98	0.345

Pairwise comparison analysis for compressive and flexural strength indicated no significant difference between the 10% and 5% groups in both pre-simulation and post-simulation scenarios (*P *> 0.05). Furthermore, all groups displayed no significant changes in compressive strength, flexural strength, and microhardness before and after thermocycling for all the modified groups (*P *> 0.05) (Table 2).

**Table 2:** Pairwise comparison of strength and microhardness of green-mediated nanocomposite (Ch-Ti-Zr-HA) modified GIC and conventional GIC before and after thermocycling. ^*^P-value was significant at 0.05. The *P*-value was derived from the multiple comparison Tukey HSD test. GIC, glass ionomer cement; HSD, honestly significant difference; Ch-Ti-Zr-HA, chitosan, titanium, zirconia, and hydroxyapatite

Pairwise comparison	Compressive strength		Flexural strength		Microhardness	
Thermocycling	Before	After	Before	After	Before	After
Control vs. 3%	0.001*	0.001*	0.001*	0.001*	0.001*	0.001*
Control vs. 5%	0.001*	0.001*	0.001*	0.001*	0.001*	0.001*
Control vs. 10%	0.001*	0.001*	0.001*	0.001*	0.001*	0.001*
3% vs. 5%	0.001*	0.001*	0.070	0.045*	0.001*	0.001*
3% vs. 10%	0.001*	0.001*	0.001	0.001*	0.001*	0.001*
5% vs. 10%	0.101	0.241	0.440	0.347	0.211	0.091

## Discussion

Due to its favorable physical and mechanical characteristics, GIC is commonly employed in permanent dental restorations. The utilization of nanotechnology in dentistry, particularly the integration of nanosized particles, has been investigated for caries prevention and reinforcement of polymeric composites [12]. Recent studies have emphasized augmenting the mechanical properties of dental restorative materials like resin composites by incorporating nanosized particles or nanoclusters. The incorporation of HA, which influences the bonding reaction mechanism and the formation of polysalt bridges in GIC, contributes to the improvement of mechanical properties [13]. The incorporation of HA, which influences the bonding reaction mechanism and the formation of polysalt bridges in GIC, contributes to the improvement of mechanical properties [13]. In addition, the incorporation of TiO_2_, which was selected for its chemical stability, biocompatibility, and nontoxicity [14], further improves the properties of the modified GIC in this study. The results of this study showed that the control group (conventional GIC) exhibited the lowest compressive strength compared to the experimental groups. The inferior mechanical properties of GICs are attributed to their inherent brittleness and potential weaknesses in adhesion between multiple components. In simpler terms, if the interfacial tension between components is high, this can lead to a deterioration in mechanical properties. The incorporation of nanoparticles capable of reducing interfacial tension or improving adhesion between components is therefore promising for increasing overall mechanical performance.

In a study, Sharafeddin et al. investigated the effects of incorporating micro- and nanoparticles of HA into GIC and found improvements in mechanical properties such as compressive strength, microhardness, and biaxial flexural strength [15]. Similarly, another study investigated the physical properties of TiO_2_ nanoparticle-enriched GIC, which resulted in a significant improvement in Vickers microhardness, flexural strength, and compressive strength [16]. These results are in agreement with the results of our study. Moshaverinia et al. demonstrated that cement enriched with nano-HA-fluorapatite exhibited higher compressive strength, higher tensile strength, and higher flexural strength compared to conventional GIC [17]. In a previous study, GIC modified solely with nanoparticles and a cellulose nanocomposite showed higher stress resistance compared to conventional GIC [18,19]. Studies by Moradian et al. showed that the addition of cellulose nanocrystals in GIC improved the compressive and diametral tensile strength [20]. Allam et al. added 5% chicken eggshell powder to conventional GIC, which resulted in better mechanical properties [21]. Previous studies by Kheur et al. [6] and Bali et al. [22] showed that HA nanoparticles have a morphology similar to human enamel and can effectively improve the mechanical properties of GIC. These findings are consistent with the results of the present study. Singer et al. [23] reported that GIC modified with plant extract improved flexural strength compared to conventional GIC, which supported our study in which plant-based nanoparticles were incorporated into GIC. This improvement is attributed to the chemical composition of the plant, which improves cross-linking, thereby strengthening the mechanical properties of the cement. In the study by Showkat et al., a significant difference in compressive strength was observed, indicating the superior performance of GIC modified with titanium and nano-HA, which was consistent with our study. In addition, GIC modified with TiO_2_ nanopowder exhibited the highest flexural strength [13]. Further studies confirmed these results and indicated lower compressive and flexural strength in conventional GIC [24,25]. In a more recent study by Moheet et al. investigating the hardness of conventional GIC compared to GIC with 10% nano-HA silica, improved microhardness was also observed, which is consistent with the results of our study [11]. The improved compressive and flexural strength observed with nanocomposite-modified GIC can be attributed to the nanoscale particle size, which enables effective pore filling and leads to increased packing density. This was consistent with the results of our study, which indicates the positive influence of nanocomposite modification on the strength properties of GIC.

Thermocycling is used as an accelerated aging method to simulate rapid thermal fluctuations and evaluate hydrolytic and thermal changes in materials. In our study, samples were subjected to 30,000 thermocycles to replicate oral environmental conditions. This aging process is crucial for evaluating the strength properties of restorative materials. The results showed that the compressive and flexural strength of conventional GIC decreased significantly after thermocycling. However, it was found that thermocycling did not cause a reduction in compressive strength in nanoparticle-modified GIC materials, which is consistent with previous research findings [26]. Another study by Souza et al. reported that incorporating nanoscale inorganic particles such as alumina or zirconia can augment the mechanical attributes of GIC following thermocycling [27]. Moreover, the SEM images after thermocycling showed smooth surfaces without microcracks in the 5% modified glass ionomer samples. The topography of the 3% modified sample showed no significant differences after thermocycling. In contrast, significant cracks and fractures were observed on the surface of the conventional GIC after thermocycling. It is important to recognize the limitations of the study, including the inability to accurately replicate the clinical conditions in the test environment. Variables such as the clinical environment, moisture contamination, and mixing techniques can affect the physicomechanical properties of dental cement. In addition, factors such as acidic environments could influence the results, highlighting the need for further research to thoroughly evaluate the long-term stability of the material. In this study, a novel GIC modified with a nanocomposite (Ch-Ti-Zr-HA) is presented with superior mechanical properties and SEM morphology, making it a promising material for restorative dentistry. The modification of GIC powder with the nanocomposite proved to be more successful in improving the mechanical properties. Despite the promising synergistic effect of the green-mediated nanocomposite-modified GIC, further clinically oriented studies are recommended. 

## Conclusions

In the context of the present in vitro study, it is noteworthy that the 5% and 10% concentrations of green-mediated nanocomposite-modified GIC exhibited higher compressive strength, flexural strength, and microhardness compared to conventional GIC. The application of thermocycling as an in vitro aging procedure possibly influenced the mechanical properties of conventional GIC restorations. SEM images underlined the significance of these results and showed significant changes on the surface of the conventional GIC samples after thermocycling treatment. This comparative analysis sheds light on the improved mechanical performance of the green-mediated nanocomposite GIC formulations and indicates their potential advantages over conventional GIC in terms of compressive strength, flexural strength, and microhardness under thermocycling conditions.
